# Pharmacist Perceptions and Future Scope of Telepharmacy in New Zealand: A Qualitative Exploration

**DOI:** 10.1155/2024/2667732

**Published:** 2024-11-14

**Authors:** Hina Saeed, Shane Scahill, Julie Kim, Raevienne Moyaen, Dharini Natarajan, Ayaka Soga, Manvis Wong, Nataly Martini

**Affiliations:** School of Pharmacy, The University of Auckland, Private Bag 92019, Auckland 1142, New Zealand

**Keywords:** Clinical Adoption Framework, pharmacists' perceptions, telehealth, telepharmacy

## Abstract

**Background:** Telepharmacy is the delivery of pharmacy services through telecommunications technology when patients and pharmacists are separated by physical distance. Falling under the broader term of telehealth, telepharmacy has been adopted globally and proved invaluable during the COVID-19 pandemic.

**Objective:** The study is aimed at investigating New Zealand pharmacists' perceptions of telepharmacy adoption and assessing its potential impact on their future scope of practice.

**Methods**: New Zealand registered pharmacists were recruited through convenience sampling to participate in semistructured interviews conducted between July and August 2022. Interviews were transcribed and coded with NVivo and analyzed using inductive thematic analysis to develop themes. Themes and subthemes were mapped to the Clinical Adoption Framework (CAF) to interpret the findings.

**Results**: From 23 participants, 70% were community pharmacists. The COVID-19 pandemic accelerated telepharmacy adoption in New Zealand and most pharmacists perceived it as convenient and beneficial to increase access to pharmacy services. Barriers such as the lack of resources, technology issues, inadequate remuneration, and existing legislation were highlighted, as was the need for better staff training, patient education, better access to technology, regulatory reforms, and integration with existing services.

**Conclusion:** There is a need for reforms and initiatives for sustainable and equitable telehealth use in New Zealand. While preparing for digital infrastructure and capabilities presents challenges, this investment can transform pharmacy practice in the long term, benefiting both patients and pharmacy professionals.

## 1. Introduction

The integration of information and communication technologies into healthcare delivery has fueled the rise of telehealth services, a trend that has accelerated in recent years. Telehealth, as defined by the New Zealand Telehealth Forum, involves using these technologies to deliver healthcare services when patients and care providers are physically separated by time or distance [1]. Telepharmacy, a specialized branch of telehealth, focuses on providing pharmacy services remotely, addressing key issues such as pharmacist shortages and limited healthcare access in rural areas [2, 3]. The importance of telepharmacy was reinforced during the COVID-19 pandemic, as remote healthcare became essential due to restrictions on in-person services [4].

Global studies highlight telepharmacy's effectiveness in diverse healthcare environments. In the United States, telepharmacy services in primary care reduced hospitalization rates [5], while in Australia, virtual pharmacy services in rural hospitals enabled uninterrupted care during the pandemic [6]. Similarly, Saudi Arabia's telepharmacy anticoagulation clinics proved as effective as in-person consultations, with high patient satisfaction and no significant clinical complications [7].

In New Zealand (NZ), advancements in technology and connectivity have accelerated telehealth adoption over the past decade [8], with the Pharmacy Council—responsible for registering pharmacists and setting standards—emphasizing the importance of pharmacists adapting their professional responsibilities to accommodate telepharmacy's expanding role [9]. This is increasingly important as NZ pharmacists provide extended health services in addition to their core duties of dispensing medications and offering over-the-counter care. These services, outlined in Table 1, require pharmacists to meet specific educational and training requirements as defined by the Pharmaceutical Society of NZ Pharmacist Services Framework [10].

To accelerate and expand telepharmacy, assessing demand for these services is essential while addressing the key facilitators and barriers to their implementation. These barriers can be categorized as structural, clinical, and patient centered [11]. Structural barriers include legal issues such as licensure, credentialing, liability, reimbursement, and telehealth infrastructure costs [11, 12]. Clinical barriers involve maintaining patient–provider relationships, upholding the standard of care, obtaining informed consent, and safeguarding privacy and security. Patient-centered barriers involve access to technology, reliable broadband, digital literacy, and connectivity issues [13]. Addressing these obstacles is essential for the broader adoption of telepharmacy.

Moreover, the success of telepharmacy depends on its acceptance by health professionals and the broader community [14]. Telepharmacy implementation in NZ is fragmented and dependent on available facilities and technology, with no comprehensive investment to date. This research is aimed at investigating NZ pharmacists' perceptions of telepharmacy adoption and assessing its potential impact on future scopes of practice. It seeks to identify key factors influencing telepharmacy integration into NZ's healthcare systems, along with the challenges and opportunities it presents.

## 2. Methods

Ethics approval for this research was granted by the University of Auckland Human Participants Ethics Committee (reference number: 24437). The study is reported according to the Standards for Reporting Qualitative Research (SRQR) guidelines [15].

### 2.1. Study Design

A qualitative approach using semistructured interviews was used to address the aims of this study, grounded in a critical realist epistemology. Critical realism acknowledges an objective reality while recognizing that our knowledge of it is shaped by social, cultural, and historical contexts. This epistemological stance supports the methodological approach, allowing exploration of both observable phenomena and the underlying mechanisms that influence them [16]. The interview schedule was adapted from Kayyali et al. [17] to fit the scope of the study. The schema explored participants' awareness of telepharmacy and their personal experiences, including perceived benefits, barriers, and potential for telepharmacy in NZ. A shared definition of telepharmacy was provided to ensure clarity and context, enabling participants to respond based on their practice insights.

The definition used is as follows: “A virtual health care delivery system where pharmacy services are provided by a pharmacist when separated by physical distance using technology such as video calls and telephones. The pharmacy services include, but are not limited to, medicine reviews and reconciliations, checking drug dispensing, patient consultations and counselling, patient monitoring, verification of compounding, medication management, and clinical assessments. Furthermore, in our definition, we consider telepharmacy as telehealth in a pharmacy setting.”

Pilot interviews were conducted with six participants, including two pharmacy students, two interns, and two practicing pharmacists. The composition of questions, the question order, and the participant's comprehension of the questions were evaluated. In response, additional prompts were added to enhance clarity, and some questions were removed or reorganized to improve the interview flow. Pilot interviews were excluded from the dataset.

### 2.2. Study Population and Recruitment

Registered and practicing pharmacists were recruited through convenience sampling. Invitations to participate were advertised in the Pharmaceutical Society of New Zealand (PSNZ) weekly email newsletter, the New Zealand Community Pharmacy Facebook group, with 4200 members, and LinkedIn. Invitations were distributed by email to hospital departments via the New Zealand Hospital Pharmacist Association (NZHPA) network. A participant information sheet accompanied the invitation via a link, detailing the research background and rationale and providing the researchers' contact information. Those interested in participating contacted a designated researcher in the team, who coordinated interview scheduling based on mutual availability. A sample size of 20 was considered adequate for this qualitative study to achieve data saturation and capture a broad range of views [18].

### 2.3. Data Collection

Once participants agreed to the interview and consented, demographic data were collected from participants via an online questionnaire, which was emailed to them prior to the interview. Interviews were conducted online via Zoom videoconferencing between July and August 2022 and recorded with the participants' prior approval. The audio recording was saved for later transcribing. Each interview was conducted by one student from a team of five undergraduate pharmacy student researchers. Student researchers received prior training from supervisors on qualitative interviewing techniques and participated in mock interviews to ensure they were confident with the whole interview process. Pharmacists were provided with a NZ $30 gift voucher as a token of appreciation for their time.

### 2.4. Data Coding and Analysis

Interviews were transcribed verbatim by a research team member and reviewed for accuracy by each interviewer after which the transcripts were uploaded to NVivo 13 (2020, R1) for analysis.

An initial exploratory phase of data analysis was employed where data were analyzed using general thematic analysis [19]. This approach allowed the team to remain open to the development of unexpected or new findings that were not defined by any predetermined deductive framework to begin with. This iterative process involved each researcher reading through their respective transcripts to familiarize themselves with the data, generating initial codes, searching for themes, reviewing and refining these themes, and ultimately, defining and naming them. To ensure the integrity of the analysis, a research fellow (H.S.) independently reviewed all data using the same methodology, which a senior team member (N.M.) reviewed to confirm that the overarching themes accurately represented the data. The deductive phase was then employed to refine the themes developed.

Several models and theories have been developed to explain user adoption of new technologies, highlighting factors that influence user acceptance, such as the technology acceptance model [20], theory of planned behavior [21], diffusion of innovation theory [22], theory of reasoned action [23], model of PC utilization [24], motivational model [25], and the unified theory of acceptance and use of technology [26]. For this study, the Clinical Adoption Framework (CAF) was used to organize and interpret the findings after data collection. This framework, designed specifically for clinical settings, evaluates health information technology adoption within healthcare organizations [27]. It accounts for the complexities of healthcare environments, including clinical workflows and patient care, and provides a multilevel analysis of telepharmacy implementation across individual users (micro), organizations (meso), and broader healthcare systems and policies (macro). The CAF's comprehensive, multistakeholder approach, encompassing patients, clinicians, and policymakers, makes it well-suited for telepharmacy adoption. Further details on the framework categories and their definitions can be found in Table S1 of the supporting information.

To ensure the validity of aligning our themes with the CA Framework, the team revisited the coded data, juxtaposing our original themes and subthemes with the elements of the framework. This not only provided a structured way of presenting the data but also enabled deeper insights into the context and relevance of the findings in the broader clinical landscape. Differences in the coding were resolved through discussion between senior team members. An ellipsis was used to remove filler words such as “um,” ah,” “hmm,” “like,” and “you know.” Square brackets were used to replace words such as “it” for clarity in direct quotes.

## 3. Results

Twenty-three pharmacists agreed to participate in the study. Of these, the majority were female (*n* = 16; 69.6%), under the age of 40 (*n* = 19; 82.6%), and of Asian ethnicity (*n* = 16; 69.6%). The majority had been in practice for less than 10 years (*n* = 15; 65.2%) and were working full-time or part-time in a community pharmacy (*n* = 16; 69.6%) in a major NZ city (Table 2).

The themes and subthemes identified from the interviews were mapped to the micro, meso, and macrolevels of the CAF as below.

### 3.1. Microlevel

#### 3.1.1. Quality

Pharmacists expressed concern regarding the functionality of video communications and described it as challenging and demanding due to their infrequent use of these systems. The initial set-up time and technical glitches impacted the workflow, resulting in fewer patient consultations. Pharmacists believed that video-based telepharmacy services would be more readily embraced if use became more widespread and familiar.

“By the time [video communications] becomes, like an everyday thing, maybe like telephones sort of thing, then maybe it will be easier. But if it's not for everybody, then the time to set up and people will want to be fiddling around and you know, actually is impacting on clinic flow, and the number of patients that we can see... But if everybody's on Zoom or telehealth or you know, people can't hear or whatever, and people are taking extra time, then it will limit the number of patients that we can actually see on a day.” (P16 Hospital Pharmacist)

Pharmacists raised cybersecurity and data privacy concerns with online technologies, stressing the importance of exploring and implementing effective measures to safeguard sensitive patient health information.

“The technology cybersecurity, how is all this protected? I know Zoom has some sort of like encryption service, and so on and so forth. But you really do have to think about cybersecurity; how is all this going to be kept safe?” (P8 Community Pharmacist)

“In terms of privacy, I think there are issues with some free online video calling services that, you know, they're out there to mine data for big corporations. So I think you do have to be careful with what platform you use.” (P15 Hospital Pharmacist)

#### 3.1.2. Use and User Satisfaction

The level of telepharmacy exposure and utilization varied among participants. While most pharmacists were familiar with the concept, over half had actively used it in their practice. However, experience and usage varied with pharmacy business models. In larger chain pharmacies, telepharmacy use was minimal, a situation attributed to the higher-paced nature of their work environment.

“...highly unlikely we will ever call a patient and like counsel and do something because, the business model for them matches just pumping out prescriptions and doing as much counselling as you can like while they're in person. But in the smaller pharmacy where service is very heavily emphasized, what we do is the patient will be started on new medication then we will touch base with them in two to four weeks and call them and ask them like, what they're feeling.” (P3 Community Pharmacist)

Pharmacists used various telecommunication media for telehealth services, including telephone calls, video conferencing (e.g., Zoom™), text messaging, faxes, and emails. The choice of medium depended on the practice setting, with community pharmacists mainly using the telephone, while primary health organizations and hospital pharmacists favored video conferencing for patient education and multidisciplinary meetings, especially during the COVID-19 pandemic.

“We were doing a lot of Zoom meetings for our multidisciplinary team meeting. So often in the hospital, pharmacists have meetings with, like doctors and dietitians, and nurses, and we kind of sit down together.” (P22 Hospital Pharmacist)

During the pandemic, telepharmacy services expanded to include remote consultations, management of long-term conditions, medical chart reviews, responses to clinical queries, communication of treatment changes, and facilitation of prescription refills. Most pharmacists were satisfied with telepharmacy during this time and hoped for its continued use and expansion.

“I hope that [telepharmacy] will continue, I hope that it will expand and be able to be used in a lot more different way than we are now. Because we were barely using it before COVID, and now it's expanded so much. So, I hope that we can find more ways where we can keep on using it and I think it'd be really helpful for patients too.” (P12 Community Pharmacist)

#### 3.1.3. Net Benefits

Most pharmacists believed telepharmacy could improve access to healthcare where in-person visits were challenging or impossible, for example, mobility limitations, COVID-19 isolation, or constraints with travel, and provide convenience, cost, and time savings.

“It is convenient for those with mobility issues, and other issues like those who can't come to the pharmacy [due to] COVID symptoms, nasal symptoms or sneezing. You can recommend something to them so you can technically still provide health care while they are [physically] not there.” (P17 Community Pharmacist)

Some pharmacists believed that telepharmacy offered comparable interaction to in-person consultations, benefiting shy patients who might find it easier to discuss their health issues virtually. Telepharmacy was also seen as practical for counselling and consulting with patients while preserving their privacy. Virtual consultations could create a more comfortable and private environment for patients embarrassed to discuss certain medications.

“It's kind of the same as you would interact in a pharmacy face to face, you can still tell them the same things, do all the same consults and everything … customers or patients find it easier to ask certain things because they are not in the pharmacy.” (P12 Community Pharmacist)

“I know that [consultation rooms] are not always used in pharmacy, even if they are available, or it still makes them a bit uncomfortable, but over the phone, they can just talk to you at home…so they're a lot more open about their questions and things like that.” (P13 Community Pharmacist)

Others reported the benefits of telepharmacy in enhancing work efficiency, flexibility, and time saving by enabling the remote resolution of healthcare issues, allowing more time to engage with patients.

“It made my workload easier, rather than adding to my workload. If I can solve someone's problem over the phone, or I don't necessarily have to wait for them to come in 10 minutes before closing, to then wait, and then on top of whoever's already in.” (P2 Community Pharmacist)

However, the inability to conduct physical assessments during virtual consultations was considered a limitation. Additionally, language barriers and reliance on audio-only mediums increased the risk of miscommunication, as the absence of visual cues made it difficult to assess patient comprehension, especially for complex instructions.

“In terms of skin conditions, or something where a close-up personal look would be better than the thing showing it on camera, Skyping- but seeing the colors of special things on screen is not clear.” (P17 Community Pharmacist)

“I couldn't sort of sometimes gauge their understanding, especially when I need to give them slightly more complex instructions on what to do with the medications. I can't gauge from the tone, or sometimes I couldn't understand because I couldn't see their face. I don't know whether they understood anything. And sometimes I think through the phone, it was quite hard to catch, I guess the details sometimes. That's not quite as easy as I thought that would be.” (P16 Hospital Pharmacist)

Some pharmacists were concerned that remote interactions could result in a lack of connection and empathy, particularly when discussing sensitive topics, and could be less suitable in building patient rapport and trust compared with face-to-face interactions.

“… face to face communication has so many advantages, you know, you're able to pick up on emotion, you're able to establish rapport and show empathy. And that can be quite difficult to do using technology, particularly when you're wanting to address some patients concern, or it's quite a sensitive topic.” (P22 Hospital Pharmacist)

Pharmacists believed that certain health issues, particularly those involving complex medical needs, would always require in-person consultations. They emphasized the importance of choosing the right communication method to meet their patients' care requirements.

“And we also look at their health condition...So they need to come in for those that are comorbid or having a very big surgery. But for those that are just having like minor skin sort of surgery or minor keyhole surgeries for gallbladder removals or something like that, and they're healthy and well, they could easily be done by telehealth.” (P16 Hospital Pharmacist)

Several pharmacists noted that patients' access to telehealth services relied on them having the necessary technology, which was a barrier for those who lacked access to devices, such as smartphones, and adequate internet access. Additionally, those residing in rural areas were often out of coverage.

“If someone doesn't have access to a proper internet, like they have slow internet, or they don't have access to any [technological] device. That is, obviously the number one barrier. Because it just defeats the purpose of the whole point. Yeah, [then] they can't really do it (telehealth).” (P11 Community Pharmacy and Academia)

There were suggestions to establish centrally equipped technology centers in community hubs within resource-limited rural communities to expand remote access to healthcare for the entire community.

### 3.2. Mesolevel

#### 3.2.1. People

##### 3.2.1.1. Individuals and Groups

Pharmacists and patients were identified as the key group of stakeholders responsible for the successful adoption of telepharmacy.

“The biggest stakeholder in this is the patient, how does the patient feel. They need to have some focus group of something to maximize the experience… you know and then again you need the pharmacist's opinion as well. How will they get it?” (P10 Health IT Pharmacist)

##### 3.2.1.2. Personal Characteristics

Several pharmacists observed that certain patients, particularly those with hearing, sight, and cognitive impairments, face greater challenges with technology. While digital hesitancy and a preference for face-to-face interactions were reported across all age groups, it was more pronounced among older patients who often lack familiarity and experience with technology. Additionally, language barriers posed further limitations in effectively using telehealth services.

“There are a few times we did zoom, but I feel like people don't normally know how to use Zoom at the time. Even now, sometimes it's pretty hard for a lot of people to do Zoom.” (P16 Hospital Pharmacist)

To address some of these challenges, pharmacists proposed conducting educational training sessions for people and collaborating with cultural groups or community leaders to facilitate the adoption process.

“Having like training sessions, like sort of weekly or monthly training sessions about what it is, just educating people about this new service and how it's going to work and why it's important and how it will benefit them…and reaching out to like having collaborations with local like marae [meeting grounds] or any cultural groups as well as just liaising with the cultural leaders and making them aware that we have the service and then they can tell people that they have connections with as well.” (P8 Community Pharmacist)

While many pharmacists embraced telepharmacy, some, particularly older pharmacists, preferred in-person consultations due to the simplicity of traditional/conventional methods. There was also a noted resistance to change among peers, especially those in managerial or senior positions, who were believed to be more inclined toward traditional/conventional methods and reluctant to adopt new approaches.

“I think there's a lot of change resistance because it is a new model of delivering pharmacy. And what you have, you've got a lot of senior pharmacists that are ‘stuck in their ways' will be the words, and they're going to be change-resistant.” (P2 Community Pharmacist)

“Yeah, I think it's gonna work quite well, if we try to try to make it work. But I'm not too sure about the hospital setting. That's the main mindset the managers would have to change. I guess, if they're happy for us to do telehealth interviews more often than face to face. Yeah, currently, I think the traditional ways of working are sort of promoted by the managers.” (P21 Hospital Pharmacist)

##### 3.2.1.3. Personal Expectations

Pharmacists anticipated that telepharmacy would enhance their role by improving their capabilities and allowing them to shift their focus more on clinical aspects of patient care.

“I feel like (telepharmacy) is enabling our role and making it better and more clinical.” (P18 DHB employed pharmacist)

Participants highlighted that telepharmacy could be effectively integrated into the existing infrastructure for scheduled pharmacist-led clinics, as well as for delivery of medication use reviews and clinical consultations.

“Yeah, I think those kinds of situations where pharmacists already do schedule clinics, I think could be really helpful. Like pharmacists working with GP practices or pharmacists working in hospitals that do that kind of thing, and pharmacist prescribers. I think those are all situations where it obviously translates quite easily. It's very similar to doctor's clinic. So, it makes sense.” (P15 Hospital Pharmacist)

#### 3.2.2. Organization

##### 3.2.2.1. Strategy

Where pharmacists emphasized the importance of strategic alignment and stakeholder engagement for sustainable telepharmacy implementation, they suggested careful planning, including conducting preliminary feasibility studies to assess viability and identify potential challenges, rather than a “plug and play” approach.

“It's gonna take a lot of planning and implementation… some of the rural places where access issues, talk to the owners, go and have some patient interaction. Get their opinion. So maybe you can do like an email survey … and then setup like a server and zoom things and then get feedback.” (P17 Community Pharmacist)

While some favored a trial-and-error approach to refine and optimize the implementation process, others were keen to learn from the experiences of their peers who had already implemented telehealth, benefitting from their experience.

“I think it might be a good idea…if you talk to people from other professions like GPs or physios or podiatrists, or other professions that have already started using telehealth, you could learn like more about how they're doing at what they found was difficult. And what they found was beneficial and yeah.” (P8 Community Pharmacist)

Participants also advocated for standardized implementation procedures and practices to promote broader adoption of telepharmacy. However, they acknowledged that the widespread adoption of telepharmacy would be a gradual and complex process, particularly slower in rural areas, as commonly observed with new technologies.

##### 3.2.2.2. Culture

Pharmacists highlighted differences in the organizational culture of pharmacies, particularly regarding the preferred model of service delivery. Those in face-to-face service settings were receptive to telepharmacy if it could demonstrate benefits for patients. Conversely, those who had been using technology developed a more nuanced perspective. They recognized the clear advantages but also noted the limitations they faced while using telepharmacy.

“It's just the way we work in the pharmacy, so we provide all the services face to face. Even with the day-to-day job as a pharmacist, like dispensing, checking, everything is done, you know, on premises. The patient is waiting for their prescription to be filled. So, there hasn't really been a chance do it, rather than choosing not to do it.” (P11 Community Pharmacist and Academia)

##### 3.2.2.3. Structure and Processes

Pharmacists viewed telepharmacy role in patient care processes to ensure continuity of care by bridging gaps in healthcare services. They saw it as a tool to connect different healthcare entities, benefiting patients. They emphasized that telepharmacy could facilitate smoother transitions between different levels of care, for instance, transitioning from hospital-based care to community care, thereby enhancing the overall quality of healthcare services.

“If a person were to be discharged from hospital, and they went back to the community, sometimes patients are not aware of changes to medicines that were made in hospital or what their medicines are for. I noticed there's like a disconnect between hospital discharge and community pharmacy, and we can make that link better [with telehealth] and help improve patient outcomes.” (P8 Community Pharmacist)

Pharmacists also noted that telepharmacy improved interprofessional communication within the healthcare team. This enhanced communication was seen as a valuable asset, improving patient outcomes by facilitating the development of collaborative treatment plans.

“I've also used it where they've been at their GP, and they've asked me to zoom in and provide some advice. We put together like monitoring plans and it's been really beneficial for [patients].” (P18 DHB employed pharmacist)

##### 3.2.2.4. Info and Infrastructure

The requirement of technological infrastructure for implementing telepharmacy was raised, including essential equipment, high-speed internet access, a unified healthcare database, and user-friendly platforms. Some pharmacists highlighted the need for a centralized healthcare database for universal access to patient health information.

“First of all, like, just one database that carries that everyone can share and have access to information would be key instead of everyone doing their own thing.” (P14 Community Pharmacist Manager)

The ease of use of telepharmacy platforms was also emphasized. Accessible and easy-to-navigate interfaces were highlighted as crucial to ensure effective use by individuals with varying levels of technological expertise.

“I'm sure we could, technology is only getting better. So, I guess it's just having a sort of platform that's really straightforward for older people to use. That can set up a call set up a video call, and that sort of stuff.” (P1 Community Pharmacist)

#### 3.2.3. Implementation

##### 3.2.3.1. Project

Pharmacists raised concerns about the existing shortage in the pharmacy workforce, noting that introducing telepharmacy could also increase workload pressures.

“Currently with a staff shortage, you need someone dedicated to be answering in a separate room or consult room or something. But with the I suppose a premium on space, it's hard to set aside that. And the resources in general, the focus on that, and making people aware of that, and how we're going to advertise it, who's going to be like, responsible for maintaining it. And we, we have practically, can we put time and money and resources.” (P17 Community Pharmacist)

They also highlighted the importance of having a designated space to ensure patient privacy and effective service delivery, and the need for a robust promotional strategy to raise awareness and encourage the adoption of telepharmacy services.

Some pharmacists identified the need for training to enhance their digital skills and communication techniques specifically for telehealth services.

“We need more a little bit more education on how to communicate more effectively. Yeah, definitely a pharmacy needs a little bit of training to make it more effective.” (P4 Hospital Pharmacist)

A few suggested that universities should update their curricula to prepare future pharmacy graduates with the skills needed for the digital healthcare world.

“…it's important to educate future pharmacists on that …so when you actually graduate and become a registered pharmacist, you're not intimidated by the fact that technology is now part of it. So I guess, if it's started from the very beginning when you are a pharmacy student, that can help.” (Participant 11 Community Pharmacist and Academia)

Most saw telepharmacy as an alternative way to reach people and did not anticipate significant changes or threats to the profession. However, some predicted a division of roles, with some pharmacists focusing on in-person services and others on online delivery.

“I think job division will be big as well. So, I think there will be pharmacists that purely work on dispensing, just getting the medication made up and sent and supplied to the patient. And then pharmacists that work online, the services monitoring, and like, stuff like that, I think there'll be some divide.” (P3 Community Pharmacist)

Some pharmacists believed that the efficiency gains from online delivery could lead to staff redundancies. Others were more concerned about retail assistants, whose roles largely depend on in-store customer interactions.

“Yeah, for those retailers who sell products and cosmetics; the main day to day thing is talking to customers in the shop and helping them. Depending on how their sales are, if they are scarce, they will be a bit more worried about telepharmacy. They may consider their productivity and decide: ‘Is the pharmacy still going to need them?' or like ‘what will they do if they lose this job?'” (P17 Community Pharmacist)

Moreover, there was fear that larger pharmacy chains, with their considerable resources, could dominate the telepharmacy market, potentially threatening the sustainability of smaller, independent pharmacies.

“There will always be kind of bigger pharmacies, who can monopolise more on that because they have the extra resources compared to smaller independent pharmacies. That's kind of the only threat I could see, but not necessarily a threat to the profession itself, because we're all in the same profession, but I guess to business owners themselves.” (P1 Community Pharmacist)

##### 3.2.3.2. Practice Fit

Pharmacists noted that adopting telepharmacy would require changes to their existing processes. Most community pharmacists believed that having a pharmacist dedicated to telepharmacy would allow consultations to be scheduled in advance, offering flexibility for both pharmacists and patients. This approach could help address current challenges in community settings, where patient interactions are unscheduled and busy periods are unpredictable, often leading to increased stress levels for pharmacists.

“If people are interested then we can schedule telehealth appointments. I think that would be a relief for pharmacists that are really struggling with the workload. I think with community pharmacy, it's not just the workload, it's the way that the workload is unpredictable, and people come in whenever they want.” (P8 Community Pharmacist)

It was also noted that the use of technology would allow more flexible work arrangements, enabling pharmacists to work virtual shifts from home—a concept that gained wider acceptance during the COVID-19 pandemic.

“Because being in a community pharmacy with a shop, you always have to be there. There's no flexibility. So, it will be quite nice to rotate a pharmacist, today you get to work from home, you do telepharmacy or could be on call like: ‘pick up the phone now' so then they are fully focused on that.” (P14 Community Pharmacist Manager)

### 3.3. Macrolevel

#### 3.3.1. Governance

##### 3.3.1.1. Legislative Acts

Pharmacists identified the absence of supportive laws for telepharmacy services as a significant barrier, with most emphasizing the need for legal reforms to accommodate the evolving telepharmacy landscape.

“Some of the disadvantages, I think, are maybe from the legal aspect of it. If you're checking prescriptions and you are not on site, then the law will have to change so that...a pharmacy is not under the supervision of a pharmacist at all times. Because you're not physically there.” (P20 Community Pharmacist and Academia)

##### 3.3.1.2. Regulations and Policies

Since telepharmacy is a new modality, many pharmacists believed that a comprehensive policy framework would provide much-needed clarity on legal considerations, operational protocols, and quality assurance measures.

“Also, policies what do you have to regulate these things, it's a healthcare service, what new policies have to come into place? What law do you have? What do you have to maintain in your pharmacy to make sure you've kept up to date with the latest telepharmacy standards or the latest telepharmacy rules? How's that going to be audited? “ (P10 Health IT Pharmacist)

##### 3.3.1.3. Governance Bodies

Pharmacists' experiences during the pandemic highlighted gaps in support from governance bodies. With the introduction of new rules for electronic prescriptions to facilitate virtual care, many pharmacists found themselves frustrated by the government's lack of clear directives.

“Because they...we didn't get informed ahead of time, even from the government actually, with regards to these. It was just announced. And then we got phone calls, but we couldn't really do it until we got direct instructions from people on exactly how we're going to do it. So that was, I think, another barrier, the government made it a bit hard for us. They didn't inform us beforehand that something like this was going to happen so we couldn't accommodate for it.” (P13 Community Pharmacist)

They also struggled to adapt to the new service model, often having to create their own protocols due to limited guidelines from governing bodies. The majority agreed on the urgent need for specific guidelines to effectively implement telepharmacy, particularly in light of evolving laws.

#### 3.3.2. Funding

##### 3.3.2.1. Remuneration

The initial implementation costs associated with telepharmacy services posed a significant financial burden for many pharmacies. Most stressed the need for government funding to support the rollout of these services. Some of the NZ government provided funding during the COVID-19 pandemic to help procure essential technological devices, and even supply some patients with mobile phones or Internet credit; pharmacists highlighted the ongoing need for such support.

To ensure the sustainability of telepharmacy services, several pharmacists suggested payment compensation for providing these services. They warned that without financial support, pharmacies might prioritize other revenue-generating services, such as vaccination administration or promoting profitable retail items, over telepharmacy services.

“So I think if the government were able to take on our initiative of telepharmacy and provide funding, and you know, have a sort of fee that they give for each consultation we do in telepharmacy I think that would be a really good incentive for us to like, as community pharmacists to take that up as a service. And, yeah, I think cost is the main thing.” (P8 Community Pharmacist)

##### 3.3.2.2. Added Value

A few pharmacists emphasized the anticipated return on investment for the broader healthcare system by investing in telehealth services—the value. They recognized its usefulness in mitigating the spread of COVID-19 and believed that embracing technological solutions for other infectious conditions could potentially reduce disease transmission, leading to improved public health outcomes.

“So now we've got, you know, COVID is a is a major issue. So, and if there's any kind of contagious disease, if we can do things more, based on technology, it might just help us, you know, decrease the spread of diseases.” (P23 Community Pharmacist)

Many saw telepharmacy as a viable way to expand pharmaceutical care and the range of services available to patients. This was particularly beneficial in remote or underserved regions, where access to comprehensive healthcare services is often limited. Providing these additional services was expected to improve health outcomes in these areas.

“I work in our West Coast area. I think there are three hospitals over the West Coast area. Yeah, we are the only pharmacy in the hospital. So we cover from Karamea to Haast [distance of 510 kilometers]. So when a patient goes to other hospitals, we don't necessarily do medication reviews. So if we could do telehealth and do a medication review with the patient in a rural hospital that might be helpful. Because quite often we don't, we don't normally get to see them when they are admitted to the rural hospital. Whereas if a patient comes to our hospital, pretty much all the patients get their medication reviewed by a pharmacist in the ward.” (P4 Hospital Pharmacist)

#### 3.3.3. Trends

##### 3.3.3.1. Societal Trends

The majority of participants believed that people have become more comfortable and willing to adapt to telepharmacy as technology becomes more ingrained in everyday life. The COVID-19 pandemic was considered the catalyst for this evolution, leading to rapid and significant changes in the perception and use of technology in healthcare compared to a decade ago.

“Because telehealth is going to be a change in the model of care, essentially. We're used to it now. It's only because of COVID. But before that it was a big deal - people would be looking at you like, “Okay, why are you even talking about telehealth“, you know? If you talked about it like 5, 6, 7 years ago. But because COVID has been normalized.” (P2 Community Pharmacist)

In light of these trends, pharmacists believed telepharmacy holds considerable potential for the future in NZ.

“With future implementation, I think it's going to grow wider… COVID has greatly pushed it and it is currently growing… I am pretty sure telepharmacy will grow in New Zealand…” (P11 Community Pharmacist and Academia)

## 4. Discussion

This study explores the perceptions and experiences of NZ pharmacists about telepharmacy, particularly following its rapid adoption during the COVID-19 pandemic. The integration of telepharmacy into the healthcare landscape marks a significant shift in the delivery of pharmacy services, and, to our knowledge, this is the first qualitative study to explore telepharmacy in NZ. The findings reveal widespread acceptance and application of telepharmacy among pharmacists as well as the various challenges and opportunities that have emerged from its use. The CAF was used to organize the findings, providing a multilevel perspective on the factors influencing technology adoption within pharmacy healthcare settings.

The findings of this study underscore the substantial increase in telepharmacy adoption in NZ during the pandemic, reflecting a broader transition towards remote healthcare services that was mirrored in general medical practice nationwide [28]. This aligns with global shifts where pharmacists and other healthcare providers rapidly adopted digital service delivery methods in response to lockdowns and social distancing mandates [5, 29, 30]. The transition to virtual care was further supported by legislative adaptations, such as the implementation of electronic prescriptions, which were critical in maintaining healthcare services during the pandemic. Additionally, professional healthcare bodies responded proactively by revising or introducing new guidelines to govern the use of technology in healthcare delivery [31].

Pharmacists in this study acknowledged the role of telepharmacy in enhancing healthcare access, particularly in rural and underserved regions, a benefit corroborated by existing research which underscores the effectiveness of telepharmacy in expanding pharmacy services to these communities [32–34]. Additionally, our findings highlight that telepharmacy offers patients a degree of privacy that is particularly valued during discussions on sensitive health issues. This aligns with studies indicating that adolescents, especially, are more engaged when discussing sensitive topics via telehealth compared to in-person consultations [35–37]. However, patient privacy and security are at greater risk when patient information is transmitted and stored remotely [38]. In response, healthcare organizations and practitioners are urged to comply with local standards. In NZ, the Health Information Security Framework provides guidelines for the safe transfer and storage of health information; however, no organization in NZ currently certifies videoconferencing services as compliant with these standards [39].

Pharmacists identified several implementation challenges with telepharmacy, including issues with technology reliability, access to essential health information in a timely manner, and the complexities involved in setting up telepharmacy services. These challenges have the potential to disrupt clinic operations and negatively impact the quality of service delivery. Telehealth-based medication reviews often require more time than traditional face-to-face consultations due to the need for additional preparations by healthcare professionals and patients to ensure effective virtual appointments (Hanjani et al. [40]. Despite these challenges, research suggests that the quality of care delivered through telehealth can be comparable to that of in-person visits [41, 42]. A meta-analysis reviewing pharmacist-led telemedicine initiatives found that out of 34 initiatives, 26 had a net positive impact (Niznik et al. [43] further highlighting the importance for pharmacy leaders to consistently monitor and assess the quality of services provided as telepharmacy becomes more integrated into healthcare practice.

This study identified several patient-related barriers that could restrict access to telepharmacy services, particularly related to technological issues such as Internet connectivity. Despite recognition by the Ministry of Health in New Zealand that effective telehealth services hinge on fast broadband internet access, data from the 2018 census indicates that 13% of households still lack internet access—a figure likely underestimated due to methodological flaws in the census [44]. Consequently, while telehealth may offer enhanced access to healthcare for technologically equipped populations, it simultaneously risks intensifying existing barriers for disadvantaged groups. This dual impact of telehealth, which can both bridge and widen the healthcare access gap, is corroborated by findings from international research [33, 34, 45], highlighting the complexity of telehealth's role in exacerbating healthcare inequities.

There was considerable resistance to adopting telepharmacy among some pharmacists in this study, particularly those who are older or work in traditional pharmacies, showing a preference for in-person interactions. This reluctance often stemmed from discomfort with new technologies and a loyalty to established approaches to care. Current literature on the impact of demographic factors such as age or experience on pharmacists' willingness to adopt telepharmacy is limited. Nonetheless, pharmacists' adoption of telepharmacy is significantly influenced by their perceived benefits of the technology, ease of use, and the availability of necessary resources and infrastructure [46]. Additionally, some pharmacists expressed hesitance towards new service models involving telepharmacy, citing concerns over potential pharmacy staff shortages. Contrary to these concerns, other studies indicate that telepharmacy has been proposed and utilized as a solution to mitigate such shortages [34, 47, 48]. The reluctance to embrace new technologies is not merely a reflection of individual preferences but stems from a complex interplay of technophobia, resource constraints, and deeply ingrained professional practices [49]. Addressing these barriers may require targeted support to ease the transition and foster broader adoption.

Pharmacists emphasized the urgent need for legal reforms and the development of standards tailored to the specifics of telepharmacy practice. Internationally, the absence of specific laws governing telepharmacy is widely recognized as a significant barrier to its implementation [47, 48, 50]. While most statutes in NZ are drafted to be technologically neutral, thus not specifically addressing digital health services, compliance with existing legislative requirements remains obligatory for all healthcare delivery methods. Nevertheless, it is essential for legal and regulatory frameworks to adapt and evolve in response to the rapid technological advancements in healthcare, ensuring that they adequately support and regulate new practices like telepharmacy.

This study highlights the need for enhanced training and educational resources to improve pharmacists' capabilities in effectively delivering telepharmacy services. This includes bolstering digital literacy and familiarity with telepharmacy platforms. Research has shown that staff training and practical experience are crucial for the successful implementation of telepharmacy services [32, 34]. In response, the Digital Health Association of New Zealand has called on the government to establish a digital health academy to support the digital skills and literacy of the country's health workforce. Concurrently, implementing strategies to improve consumers' ability to use digital health services is likely to enhance their overall experience and satisfaction with telepharmacy.

Despite various challenges, pharmacists in this study believe that telepharmacy holds significant potential for growth and evolution in the future. There is optimism about its capacity to transform pharmacy practice by incorporating more digital tools and platforms into healthcare delivery. Recent studies have highlighted key factors necessary to ensure the long-term sustainability of telehealth, including developing a skilled workforce, empowering consumers, reforming funding mechanisms, enhancing digital ecosystems, and seamlessly integrating telehealth into routine pharmacy care [51]. These elements are essential for building a robust telepharmacy infrastructure that can adapt to future healthcare demands.

### 4.1. Implications for Practice and Education

The effective use of telehealth in pharmacy practice depends on the capabilities of both pharmacies and their patients. Clear communication from pharmacists about telehealth expectations, functions, and costs is recommended. Re-evaluating pharmacy practice funding to support telehealth is essential. This includes providing adequate funding to address barriers and developing innovative solutions to enhance technology access and digital literacy, especially for older adults and individuals with disabilities.

For pharmacy practices, prioritizing technological preparedness is key. This involves developing comprehensive telehealth protocols and conducting targeted training for pharmacy teams to maintain service quality. Successful telehealth implementation also requires seamless integration with existing pharmacy services to ensure continuity of care and strengthen patient relationships. This includes enhancing interoperability between health information systems and focusing on reducing health disparities.

Pharmacy practice in NZ has shown considerable flexibility and adaptability by incorporating telehealth during the early stages of the COVID-19 pandemic. Learning from this experience is vital to establishing adequate funding and operational processes. This will enable telehealth to be both a critical tool during future pandemics and a standard part of everyday pharmacy care, fostering a more resilient and accessible healthcare system.

### 4.2. Future Research Areas

Further research is essential for enhancing the integration of telepharmacy into healthcare systems and for understanding its impact on patient healthcare outcomes and patient and practitioner satisfaction. Additionally, exploring how telepharmacy can improve access to healthcare for underserved populations, such as those in rural areas, lower socioeconomic and the elderly, and Māori and Pasifika peoples, is vital for fostering equity in healthcare provision. Understanding cultural and psychological factors affecting the adoption of telepharmacy will help inform potential barriers and enablers.

Investigating the economic implications of telepharmacy is crucial, in terms of cost-effectiveness and its broader influence on the healthcare economic landscape. Simultaneously, addressing regulatory and policy challenges is fundamental to facilitating smoother adoption of telepharmacy, and developing targeted training programs and certifications is essential to equip pharmacists and pharmacy technicians to deliver telepharmacy services effectively. Moreover, fostering interdisciplinary collaboration through telepharmacy could further embed it within the broader healthcare system. Longitudinal research focused on the sustainability of telepharmacy services is critical to ensure that pharmacy continues to adapt and meet the evolving needs of healthcare systems and their patients.

#### 4.2.1. Limitations

Several limitations must be acknowledged. The use of convenience sampling may introduce selection bias, as participants could share characteristics that are not representative of the broader population, potentially skewing the results. Consequently, the findings are not generalizable to the entire population. Additionally, there is a risk of sampling bias as participants were self-selected and likely more interested in telepharmacy, which may have excluded critical views from less enthusiastic pharmacists. The predominantly young, early-career Asian demographic may not represent the broader pharmacist population, including older pharmacists, those from different ethnic backgrounds, particularly Māori, and those at various career stages. Although the study included pharmacists from diverse geographic areas, it did not thoroughly investigate the unique challenges and opportunities in less populated regions, where the impact of telepharmacy might differ significantly from urban settings. This study mainly captured the perspectives of community and hospital pharmacists, omitting those in general practice and in leadership roles within professional bodies and the government, whose insights are crucial for understanding broader systemic and policy-related factors.

Not all categories were addressed since the CA framework was used to map the data rather than generate interview questions. This indicates the need for a more integrated approach in future studies to ensure comprehensive coverage of all relevant areas. Additionally, the framework may not fully capture the complexity of telepharmacy implementation, requiring supplementary methods for a more complete analysis of telepharmacy.

## 5. Conclusion

This study has shed light on how pharmacists perceive telepharmacy and its impact on the future of pharmacy practice. Individuals' experiences and viewpoints on technology are important factors to consider when understanding pharmacists' perceptions towards telepharmacy. The benefits of telepharmacy, as perceived by pharmacists, align with existing literature. Nevertheless, this research has identified unique barriers faced by pharmacists within the NZ context. To address these challenges, the study recommends a clear, clinically-led strategy supported by the policy and an equity focus to facilitate telehealth delivery. While initial economic considerations may be associated with developing the necessary data and digital capabilities, this investment is likely to be a step towards more sustainable healthcare reforms that benefit both patients and healthcare providers. The adoption of telehealth in pharmacy practice has the potential to create a more accessible and hybrid model of pharmaceutical care in NZ, setting an example for other countries. To achieve this, pharmacists, patients, and policymakers must work together to plan and implement this transition effectively.

## Figures and Tables

**Table 1:** NZ registered pharmacists' scopes of practice.

**Category**	**Service**
Medicine management services	
Medicines adherence	
Medicines adherence Level 1	Long-term conditions (LTC): optimise supply and use of medications
Medicines adherence Level 2	Medicines use review (MUR): optimise medication understanding and adherence
Medicines optimisation	
Medicines optimisation Level 1	Medicines therapy assessment (MTA): optimise medication efficacy
Medicines optimisation Level 2	Comprehensive medicines management (CMM): optimise management of prescribed medications
Optimising specific medicines	Example: community pharmacy anticoagulation management service (CPAMS)
Health promotion and preventative services	Health education services
	Immunisation services
	Screening and intervention services
Pharmacist medicines information services	
Pharmacist-only (restricted) medicines	
Pharmacist prescribing	
Hospital clinical pharmacy services	Core clinical pharmacy services
	Medicine reconciliation, medication histories acquisition
	Prescribing in a collaborative setting
	Participation in clinical ward rounds
	Inpatient medicines chart (prescription) review
	Medicines Optimisation, Therapeutic Drug Monitoring (TDM)
	Patient medicines counselling before discharge
	Medicines and Clinical Information Support including medicines information provision and in-service education
	Medicines guideline and protocol development
	Adverse drug reaction (ADR) monitoring and management
	Medicines use evaluation (audit of medicines usage)
	Antibiotic stewardship programmes (ASP)
	Clinical research

**Table 2:** Participant demographics (*N* = 23).

	**n** ** (%)**
Sex	
Female	16 (69.6)
Male	7 (30.4)
Age	
20–29	9 (39.1)
30–39	10 (43.5)
40–49	3 (13)
6–69	1 (4.3)
Ethnicity	
Asian	16 (69.6)
NZ European	4 (17.4)
Pacific Peoples	1 (4.3)
MELAA⁣^∗^	2 (8.7)
Years in practice	
1–9	15 (65.2)
10–19	7 (30.4)
20+	1 (4.3)
Type of practice (position)	
Community pharmacy (pharmacist)	9 (39.1)
Hospital pharmacy	5 (21.7)
Community pharmacy (manager)	3 (13)
Community pharmacy and academia	2 (8.7)
Other^a^	2 (8.7)
Community pharmacist (owner)	1 (4.3)
Community and Hospital pharmacy	1 (4.3)
Work location	
Major city (population > 30,000)	14 (60.9)
Provincial city (population < 30,000)	6 (26.1)
Provincial town (population 10,000–30,000)	3 (13)

⁣^∗^Middle Eastern, Latin American, and African.

^a^Health IT pharmacist and district health board (DHB) employed pharmacist.

## Data Availability

The data that support the findings of this study are available on request from the corresponding author. The data are not publicly available due to privacy or ethical restrictions.

## Supporting Information

Supporting Information Additional supporting information can be found online in the Supporting Information section. Table S1 categorizes elements at the micro, meso, and macrolevels within the Clinical Adoption Framework (CAF), providing definitions for each. It also includes additional quotes to further illustrate these categories.
