# Supplementary material for: Pharmacist Perceptions and Future Scope of Telepharmacy in New Zealand: A Qualitative Exploration
Source: Int J Telemed Appl. 2024 Nov 14;2024:2667732. doi: 10.1155/2024/2667732 (PMC11581793; doi:10.1155/2024/2667732)
Supplement: Supporting Information — Additional supporting information can be found online in the Supporting Information section. Table S1 categorizes elements at the micro, meso, and macrolevels within the Clinical Adoption Framework (CAF), providing definitions for each. It also includes additional quotes to further illustrate these categories. [file 2667732.f2.docx]

**Supplementary Material**

Table S1: Micro, meso, and macro-dimensions, categories and definitions adapted from (Lau et al., 2011).

| **Dimension** | **Category** | **Definitions of Suggested Measures** | **Additional quotes** |
| --- | --- | --- | --- |
| **Micro Level** |  |  |  |
| HIS* Quality | Information | Content – completeness, accuracy, relevance and comprehension |  |
|  | System | Functionality – type and level of features available |  |
|  |  | Performance – Accessibility, reliability and system response time |  |
|  |  | Security – type and level of features available | *"People have to be really careful with how they use it, and also, using the right platforms…In terms of privacy, I think there are issues with some free online video calling services that, you know, they're out there to mine data for big corporations. So I think you do have to be careful with what platform you use" P15 Hospital Pharmacist* |
|  | Service | Responsiveness of the system’s support services. |  |
| Use and User Satisfaction | Use | User behavior and pattern – type, frequency, duration, location and flexibility of actual usage |  |
|  |  | Self-reported use – type, frequency, duration, location and flexibility of perceived usage | *“We use telephones on a daily basis. So customers give us a call for repeat, if they have any questions. So yeah, telephone is the one thing that we use a lot of”. P23 Community Pharmacist* |
|  |  | Intention to use – proportion of and reasons for current non-users to become users |  |
|  | Satisfaction | satisfaction - competency, user satisfaction and ease of use. | *"That [experience with telepharmacy] was really brilliant, it enabled" P18 DHB employed pharmacist* |
| Net Benefits | Care Quality | Patient safety – preventable errors, surveillance/monitoring, and risk/error reduction | *“It's a way of reaching people that you might not otherwise reach, to have those interactions… So it's not making pharmacists obsolete by technology, doesn't seem like there's any kind of threat from telehealth” P15 Hospital Pharmacist* |
|  |  | Appropriateness and effectiveness – adherence, compliance, practices, continuity of care | *"So it's yeah, it also doesn't suit a lot of patients as well. So, you know, a category of people that might not be suited. And obviously, I'm not making generalizations here because you're not supposed to...So it doesn't really suit everyone." P11 Community Pharmacy and Academia* |
|  |  | Health outcomes – clinical outcomes and changes in health status from eHealth interventions |  |
|  | Productivity | Efficiency – resource use, improvement in output, management, efficiency and capability | *"It can be time saving. Sometimes I use it when, for example, cardiology on the first floor, if I have patients who are over in another building, in ED or some other location that's really far away, I give them a call, see if I can obtain the medication history or the information I need from them. So I don't have to spend like 15 minutes to walk and just looking for them. That would be very useful. In that way, I end up seeing more patients because I save that time from walking. " P21 Hospital Pharmacist* |
|  |  | Care coordination – care provision by team and continuity of care across the continuum | *“...telepharmacy has huge scope to improve transition of care. ...seen it with the rollout of Indici^TM^, which is the prescribing system we use at our hospital for doing electronic prescriptions. The beauty is that it is like a space where prescribers can write a comment to the pharmacist. And I think that just allows for improved communication and the note stays with the prescription.” P22 Hospital Pharmacist* |
|  |  | Net cost – monetary avoidance, reductions, actual/projected savings | *"So like, some people who are like low decile may not even be able to afford transport to get into like the pharmacy, that, like the specific cases where they only have enough petrol money, which is really unfortunate to like, go to the pharmacy once a month. Like even that is like a financial barrier. So it cuts down on the financial barrier" P3 Community Pharmacist* |
|  | Access | Ability to access service – availability, diversity, timeliness and consolidation of services | *“Well, a lot of people that I work with don't have phones, or internet, or any of those things. Yeah. So obviously, they were not going to have the advantage of telehealth, which means it can be unequitable.” P18 DHB employed pharmacist* |
|  |  | Patient/caregiver participation – self-management and access to own information |  |
| **Meso Level** |  |  |  |
| People | Individuals and Groups | Types of individuals/groups who can affect the adoption of HIS, including patients/clients and families, healthcare providers and managers, policy planners, and stakeholder groups |  |
|  | Personal Characteristics | The degree to which an individual's age, gender, education, experience and expertise can affect the adoption of technology | *"And a lot of older people are very amazing with their technology, but a lot of them aren't as well. " P20 Community Pharmacist and Academia* |
|  | Personal Expectations | The degree to which an individual believes HIS is important, can improve job performance, and infrastructures exist to support its adoption |  |
|  | Roles and Responsibilities | The position, function and obligation of an individual/group in relation to HIS adoption, for example, being a stakeholder, leader, champion and project sponsor |  |
| Organization | Strategy | A set of coordinated activities designed to achieve the overall mandate and objectives of the organization, including HIS adoption |  |
|  | Culture | The ingrained set of shared values, beliefs and assumptions acquired by members of an organization over time, including their views toward HIS |  |
|  | Structure and Processes | Organizational functioning, including governance, configuration, reporting relationships, communication, as well as business and patient care processes such as continuity of care |  |
|  | Info and Infrastructure | HIS governance/management, technical architectures, information assets, level of integration and privacy/security in place or planned | *“I think one of the big things will be for community pharmacies to have better access to health information, health records, like GP information. It is a bit of a block there, which can sometimes slow down dispensing processes. So hopefully, we will develop something like electronic health records.” P22 Hospital Pharmacist* |
|  | Return on Value | Economic return on HIS investment in terms of cost benefit, effectiveness, utility and avoidance; business case, return on investment, value propositions, benefits realization |  |
| Implementation | Stage | HIS adoption stages from initiation, build/buy, introduction to adaptation |  |
|  | Project | The planning, activities and resources for HIS adoption, including scope, objectives, constraints, targets, governance, methodology, commitment, communication, training, risks, monitoring, reporting and expectations | *“You have to get everybody on board: patients, GPs, pharmacists, nurses, you have to let them know well ahead of time what's about to happen, you have to have training sessions, you have to have practice runs, you have to have information about what happens if things go wrong, or the connection drops, who do you contact, you have to lay the groundwork for this intervention before you go live with it” P10 Health IT pharmacist* |
|  | HIS-Practice Fit | The degree of fit between the HIS and organizational work practices, and the extent of change from HIS adoption | *"I think some things are going to need either need to change or continue to need to be tweaked with the way that we are practicing pharmacy now to allow a service like that to for the benefits outweigh the inconveniences so to speak." P20 Community Pharmacy and Academia* |
| **Macro Level** |  |  |  |
| Governance | Legislative Acts | The types of HIS related legislative acts, such as health information and privacy laws that govern the adoption of HIS |  |
|  | Regulations and Policies | The types of HIS related regulations/policies, such as data access and security/privacy guidelines |  |
|  | Governance Bodies | The types of accountability and decision-making structures in place regarding the adoption of HIS | *"And pharmacies have had to generate their own protocols on how they do it. I think the same with when COVID happened, like, you know, putting up those directions and two person per pharmacy or whatever. All the pharmacists did it themselves, pharmacy managers; they organized it all themselves. We didn't get too many guidelines. So yeah, it would be nice if we could get very specific guidelines on how things can be done" P13 Community Pharmacist* |
| Standards | HIS Standards | The types of data, messaging, terminology and technology standards that influence the healthcare industry as a whole with respect to HIS adoption |  |
|  | Performance Standards | The types of organizational performance standards in place such as those for accreditation of healthcare facilities and performance targets |  |
|  | Practice Standards | The desired level of professional competency, knowledge, skills and performance in the workplace, including HIS adoption |  |
| Funding | Remunerations | The types of compensation available, such as alternative payment schemes to entice change at the individual, practice and organizational levels |  |
|  | Added Values | General expectations on the return-on-value from the adoption of HIS such as improved patient safety and access to care |  |
|  | Incentive Programs | The types of reward programs available that entice change at the individual, practice and organizational levels |  |
| Trends | Societal Trends | The general expectations of the public toward healthcare and HIS | *“With Pill Drop and Zoom Pharmacy [examples of digital pharmacies], five to ten years ago, people were absolutely appalled by the idea. Because what about old people who can't use a phone? And now, eighty year old can use phones now, they're pretty technology savvy. So yeah. As our communities, people and technology evolves; pharmacy evolves too.” P19 Community Pharmacist* |
|  | Political Trends | The general political climate toward healthcare and HIS |  |
|  | Economic Trends | The general economic investment climates toward healthcare and HIS |  |

*Hospital Information System (HIS)
